# Large A-fiber activity is required for microglial proliferation and p38 MAPK activation in the spinal cord: different effects of resiniferatoxin and bupivacaine on spinal microglial changes after spared nerve injury

**DOI:** 10.1186/1744-8069-5-53

**Published:** 2009-09-22

**Authors:** Marc R Suter, Temugin Berta, Yong-Jing Gao, Isabelle Decosterd, Ru-Rong Ji

**Affiliations:** 1 Pain Research Center, Department of Anesthesiology, Brigham and Women's Hospital and Harvard Medical School, Boston, Massachusetts 02115, USA; 2 Anesthesiology Pain Research Group, Anesthesiology Department, University Hospital Center and University of Lausanne, CH-1011 Lausanne, Switzerland; 3 Department of Cell Biology and Morphology (DBCM), University of Lausanne, CH-1005 Lausanne, Switzerland

## Abstract

**Background:**

After peripheral nerve injury, spontaneous ectopic activity arising from the peripheral axons plays an important role in inducing central sensitization and neuropathic pain. Recent evidence indicates that activation of spinal cord microglia also contributes to the development of neuropathic pain. In particular, activation of p38 mitogen-activated protein kinase (MAPK) in spinal microglia is required for the development of mechanical allodynia. However, activity-dependent activation of microglia after nerve injury has not been fully addressed. To determine whether spontaneous activity from C- or A-fibers is required for microglial activation, we used resiniferatoxin (RTX) to block the conduction of transient receptor potential vanilloid subtype 1 (TRPV1) positive fibers (mostly C- and Aδ-fibers) and bupivacaine microspheres to block all fibers of the sciatic nerve in rats before spared nerve injury (SNI), and observed spinal microglial changes 2 days later.

**Results:**

SNI induced robust mechanical allodynia and p38 activation in spinal microglia. SNI also induced marked cell proliferation in the spinal cord, and all the proliferating cells (BrdU+) were microglia (Iba1+). Bupivacaine induced a complete sensory and motor blockade and also significantly inhibited p38 activation and microglial proliferation in the spinal cord. In contrast, and although it produced an efficient nociceptive block, RTX failed to inhibit p38 activation and microglial proliferation in the spinal cord.

**Conclusion:**

(1) Blocking peripheral input in TRPV1-positive fibers (presumably C-fibers) is not enough to prevent nerve injury-induced spinal microglial activation. (2) Peripheral input from large myelinated fibers is important for microglial activation. (3) Microglial activation is associated with mechanical allodynia.

## Background

Injuries to peripheral nervous system can result in neuropathic pain and contribute to chronic post-operative pain [1]. Current treatments for persistent post-operative pain are not satisfactory and prevention at early stage might be important for the success [2]. Section of a peripheral nerve induces injury discharges at the time of injury followed by spontaneous activity in the axons and soma of primary sensory neurons. The onset of spontaneous activity is strongly implicated in the generation of neuropathic pain [3-6]. However, the relative contribution of different types of primary afferents to the genesis of spontaneous activity is still under debate. Many studies demonstrated that A-fibers are the principal contributors of ectopic firing from the periphery following nerve injury [7-11]. Some studies also reported spontaneous activity in C-fibers but at different times after nerve injury, either very early during the first 15 minutes [12] or later after a few days [13]. The C-fibers' activity was also found in the neighbouring intact spinal nerve after spinal nerve ligation [5] or after stimulation of a nerve stump with nociceptive mediators [14]. Interestingly, Sun et al. demonstrated a strong correlation between ectopic discharges and pain related behavior at the early but not late phase of nerve injury [15].

Increasing evidence suggests that spinal microglia play an important role in neuropathic pain sensitization [16-18]. Microglia comprise around 5-20% of the glial cells and are of monocytic origins therefore sharing many molecular markers with macrophages. Microglial activation is described in various ways, such as changes in morphology (from ramified to amoeboid), gene expression (e.g., MCH I and II, CD 11b, Iba1), function (phagocytosis), or number (proliferation) [19]. Microglial proliferation is rarely seen in the resting or surveying state [20] but dramatically increases after nerve injury [21,22]. Recent studies have also shown that (1) nerve injury activates p38 mitogen-activated protein kinase (MAPK) in spinal microglia, (2) spinal infusion of p38 inhibitor attenuates neuropathic pain symptoms such as mechanical allodynia [16,23,24], and (3) blocking peripheral activity from the site of injury with bupivacaine microspheres prevents but does not reverse p38 activation in spinal microglia after spared nerve injury [25].

The side effects of long-term and complete nerve block, such as motor impairment, cannot be tolerated in patients. Therefore the concentration of local anesthetics is often reduced to block nociceptive fibers in the postoperative phase. Long-term and selective blockade of nociceptive fibers is attractive and can be achieved using the sodium channel blocker QX-314 combined with capsaicin [26] or resiniferatoxin (RTX), an ultrapotent agonist for transient receptor potential vanilloid subtype-1 (TRPV1) that is only expressed in nociceptors [27,28]. Nociceptive-specific block can provide analgesia without affecting motor function or pain-unrelated sensory function [29,30]. Recently electrical stimulation at C-fiber intensity has been shown to induce microglial changes [31], but it is unclear whether blocking nociceptive fibers alone would suppress spinal microglial activation after nerve injury. We set out to compare the effects of a general block using bupivacaine-loaded microspheres with a selective block of nociceptors using RTX on microglial activation in the spared nerve injury (SNI) model of neuropathic pain. To examine microglial activation, we investigated p38 activation and cell proliferation in the spinal cord.

## Methods

### Animals

Experiments were done on Sprague-Dawley rats (Charles River, MA, USA), weighing 220-250 grams. Rats were housed in the same room at constant temperature and a 12/12 dark/light cycle and had ad libitum access to water and food. The Harvard Medical School Animal Care Committee approved all animal procedures in this study.

### Drugs

5-bromo-2-deoxyuridine (BrdU) was purchased from Sigma, and prepared at a concentration of 20 mg/ml in 0.007 N NaOH and 0.9% NaCl [21]. Resiniferatoxin was purchased from Sigma and dissolved in dimethyl sulfoxyde (DMSO, 1 mg/ml) and the final concentration was 0.01% with 0.3% Tween 80, 10% DMSO, and 0.9% NaCl. The bupivacaine-loaded microspheres were kindly provided by Dr. Charles Berde from Children's Hospital, Harvard Medical School. The microsphere solution contained: 75% wt/wt bupivacaine, 24.95% wt/wt polylactic-coglycollide polymer, and 0.05% wt/wt dexamethasone [32].

### Surgery, nerve block, and group assignment of animals

Nerve block with bupivacaine microspheres: This procedure was performed as previously described [32]. Rats were anesthetized with 1.5-3.0% isoflurane, and the skin was incised from the left greater trochanter to the knee. The muscle layers were separated between the gluteus superficialis and the biceps femoralis, exposing the sciatic nerve from the emergence of the musculocutaneous branch to the trifurcation in sural, tibial, and peroneus branches. A silicone tube (11 mm long) was placed carefully around the sciatic nerve proximally from the trifurcation. Two 6-0 silk ligatures were attached around the tube to close the longitudinal slit. To facilitate the second surgery for nerve injury (see below), the common peroneal and tibial nerves were exposed and a 6-0 silk suture was passed under each nerve and left in place. A fibrin glue plug was placed at the distal end of the tube (Tissucol; Baxter, Volketswil, Switzerland), and 80 μl of solution containing microspheres loaded with 75% w/w bupivacaine (300 mg/ml) mixed in Tissucol (Baxter, Volketswil, Switzerland) fibrin sealant were then slowly poured inside the tube through the proximal end which was closed with glue. Muscle and skin were then closed in two layers, and the animals were allowed to recover. The control group had the same surgery except that sealant without bupivacaine-containing microspheres was inserted into the silicone tube.

Nerve block with resiniferatoxin (RTX): The sciatic nerve was exposed as previously described, and RTX solution (0.01%, 100 μl) was perineurally injected, followed by spared nerve injury (see below). The RTX concentration (0.01%) was based on a previous study showing that 0.01% RTX can produce thermal blockade for 2 days [33]. Control animals had the same surgery but received perineural injection of vehicle (0.3% Tween 80, 10% DMSO, and 0.9% NaCl).

Spared nerve injury: This surgery was performed at least 3 hours after bupivacaine or RTX block and after sensory/motor and radiant heat testing. The skin and muscles were reopened at the same wound. The sutures previously placed around common peroneal and tibial nerves were tightened and both nerves were cut with a 1-2 mm nerve segment was removed as previously reported [34]. Muscle and skin were closed in two layers. Special care was taken to avoid any damage to the sural nerve in all surgical procedures.

Animals were randomly assigned into 7 groups: (1) control group: including vehicle control for RTX injection (n = 3) and tube insertion control (without microsphere, n = 3), and these two groups were combined into one group (n = 6) in behavioral studies, since no differences were found between them. (2) bupivacaine alone group (tube with bupivacaine microspheres, no SNI, n = 3), (3) RTX alone group (no SNI, n = 5), (4) SNI group (with tube and SNI, without microsphere, n = 5), (5) SNI-vehicle group (n = 5), (6) SNI-bupivacaine group (tube with microspheres, n = 5), and (7) SNI-RTX group (n = 9).

### Behavioral testing

Motor and sensory conduction block testing: Rats were gently held with a cloth wrapped above their waist to restrain upper extremities, and several measurements were performed on both hind limbs. (1) Nociceptive response. The skin on both sides of the hind paws (sural and saphenous nerve territories) was pinched with a small forceps and the withdrawal reflex response (yes or no) was noted [35]. (2) Proprioception and motor function. Two additional tests were used to ensure that proprioceptive and motor functions were blocked. Briefly, hopping response is the ability of the animals, while standing on one leg with their body being moved laterally, to hop in the direction of the movement (score: 0 = no hopping, 1 = successful hopping). The tactile placing response shows the capability to reposition the paw after extension (1-4 score range: 1 is complete repositioning, 4 is no repositioning, and 2 and 3 are intermediate positions) [32,36]. All tests were performed before SNI as baseline, then on day 1 and day 2 after SNI.

Pain assessment: Baseline behavioral testing was made after 3 days of habituation to the environment and observer. To avoid the effect of the circadian cycle, all behavioral assessments were performed during the same period (08:00-11:00 AM). The investigator was not aware of the treatment applied. Treated and control animals were tested during the same session. Two baselines were taken before surgery then the animals were tested on day 1 and day 2 after SNI. To test mechanical sensitivity, animals were individually placed in a chamber on an elevated metal mesh floor and allowed to acclimate for 20 minutes before testing. Light mechanical stimuli were applied to the sural territory of a foot with a serie of von Frey monofilaments of logarithmically incrementing stiffness ranging from 0.06 g to 15 g, starting with a 2 g filament. The 50% paw withdrawal threshold (PWT) was determined using Dixon's up-down method [37]. Each filament was applied for 5 seconds and a brisk paw withdrawal was taken as positive response. To test heat sensitivity, rats were placed in a plexiglas chamber on a glass shelf. A movable radiant heat was used to stimulate the lateral part of the hind paw, and paw withdrawal latency was determined [38]. The intensity of radiant heat was adjusted to elicit a response of 10-12 s latency in normal rats (baseline) and a cut-off latency (20s) was set to avoid paw injury.

### Immunohistochemistry

To label newly synthesized DNA, 5-bromo-2-deoxyuridine (BrdU, 100 mg/kg) was intraperitoneally injected after behavioral testing on SNI day 2. Two hours after BrdU injection animals were terminally anesthetized with isoflurane and transcardially perfused with phosphate buffer saline (PBS) at room temperature followed by 4% paraformaldehyde with 1.5% picric acid in phosphate buffer (pH 7.4, 4°C). The L4 and L5 spinal cord segments were dissected, postfixed overnight in the same fixative, and then transferred to 15% sucrose PBS for cryoprotection. Spinal cord segments were frozen at -20°C in a cryostat and transverse free-floating sections (30 μm) were cut and collected in phosphate buffer.

For BrdU staining, spinal cord sections were first heated in a formamide solution containing 50% formamide, 50% 2× saline sodium citrate (2 × SSC: 175.3 g NaCl; 88.2 g sodium citrate in 1000 ml dH_2_O) for 2 h at 65°C to denature DNA. The sections were then washed 2 × 15 min in 2× SSC. To break any remaining hydrogen bonds between the nitrogenous bases of nucleic acids, sections were heated at 37°C for 30 min in 2N HCl and placed in a 0.1 M borate buffer at pH 8.5 and rinsed 3 × 10 min in Tris buffered saline (TBS, pH 7.5). Sections were then blocked with TBS containing 0.25% Triton X-100, 1% bovine serum albumine and 3% normal goat serum for 1 h at room temperature and incubated overnight with a mouse monoclonal antibody against BrdU (1:500, Chemicon, Temecula, CA) followed by a goat anti-mouse secondary antibody (1:400, Jackson ImmunoResearch Inc., West Grove, PA) for 1 hour at room temperature.

To determine if BrdU was only incorporated by microglia, we performed double immunofluorescence by combining mouse BrdU antibody with a rabbit antibody against the microglial marker ionised calcium binding adapter molecule 1 (Iba1, 1:500, WAKO) followed by a mixture of goat anti-mouse fluorescein isothiocyanate- (FITC-) and goat anti-rabbit Cy3-conjugated secondary antibodies.

For phosphorylated p38 (p-p38) staining, spinal sections were blocked with 2% goat serum in PBS containing 0.3% Triton and 0.01% NaN3. We incubated the sections overnight at 4°C degrees with rabbit primary antibody directed against phospho-p38 (rabbit, 1:500, Cell Signaling Technology, Beverly, MA). The sections were then incubated with goat anti-rabbit Cy3-conjugated secondary antibodies (1:400, Jackson ImmunoResearch Inc., West Grove, PA) for 1 hour at room temperature.

The stained sections were examined under a Nikon fluorescence microscopy (Tokyo, Japan), and images were captured with a CCD camera (SPOT, Diagnostic Instruments, USA). To obtain high-resolution images of microglia and confirm the double staining, confocal images were captured with a Zeiss LSM 510 META upright confocal microscope.

### Quantification

To quantify BrdU- and p-p38-positive cells in the spinal cord, 5-8 non-adjacent sections from the L4-L5 spinal cord segment of each rat were randomly selected, and the number of positive cells in the medial dorsal horn (laminae I-III), captured under 20× object in a box (450 × 338 μm) were counted. The examiner was unaware of the group treatment.

### Statistics

All the data were presented as mean ± SEM. Two-way ANOVA was used to assess behavioral changes over time and between treatments. Student's t-test was used to compare groups at SNI day 2. One-Way ANOVA and t-test were used to assess differences between groups for the number of p-p38 and BrdU immunoreactive cells. Significance level was set at p < 0.05.

## Results

### Different effects of bupivacaine and RTX on nociception, motor function, and proprioception

In animals receiving nerve blockade with bupivacaine microspheres, we saw impairment in performing the hop and flip test and absence of response to pinch in the sural territory. However, RTX-treated animals showed a normal sensory/motor behavior in the hop, flip and pinch test. After SNI surgery motor function was disrupted, as indicated by impairment in the hop and flip test but the sensitivity to pinch was preserved. In SNI animals the sensitivity to pinch in the sural territory was lost after bupivacaine block but preserved in the RTX-treated animals. Control animals showed a normal sensory/motor behavior in the flip, hop and pinch test. The sensitivity to pinch in the saphenous territory was preserved in all groups (Table 1).

**Table 1:** Effects of bupivacaine microspheres and RTX on nociception, motor function, and proprioception.

	**Hop**		**Flip**		**Pinch (sural)**		**Pinch (saphenous)**	
				
	**Normal**	**Blocked**	**Normal**	**Blocked**	**Normal**	**Blocked**	**Normal**	**Blocked**
				
Control (n = 6)	6	0	6	0	6	0	6	0
RTX only (n = 5)	5	0	5	0	5	0	5	0
Bub only (n = 3)	0	3	0	3	0	3	3	0
SNI+vehicle (n = 10)	0	10	0	10	10	0	10	0
SNI+RTX (n = 9)	0	9	0	9	9	0	9	0
SNI+Bup (n = 5)	0	5	0	5	0	5	5	0

Nociceptive response was tested by pinching the hind paws in the sural and saphenous nerve territories. Proprioception and motor function were examined by checking hopping and flipping responses.

### RTX blocks heat sensitivity but not mechanical allodynia after SNI

**Figure 1:**
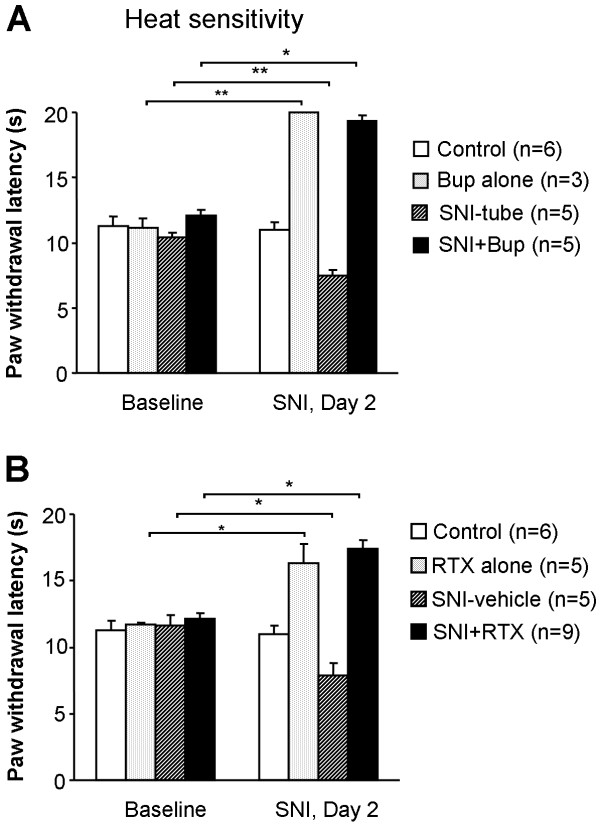
**Effects of nerve block with bupivacaine microspheres (Bup, A) and resiniferatoxin (RTX, B) on heat sensitivity before and two days after spared nerve injury (SNI)**. Both bupivacaine and RTX increase the paw withdrawal latencies in injured and non injured rats. Note a decrease in latency after SNI. Baseline pain sensitivity was tested before drug treatment and nerve injury *p < 0.05, **p < 0.01.

Two days after SNI, we observed a decrease of paw withdrawal latency to heat stimulation. The latency dropped from 10.4 s before surgery to 7.5 s (p < 0.01) in the SNI-tube group and 7.9 s (p < 0.05) in the SNI-vehicle group. Bupivacaine completely blocked heat response in all animals (with or without nerve injury) (Fig. 1A). RTX increased the paw withdrawal latency in both RTX-alone and SNI+RTX groups, compared to baseline and SNI-vehicle groups (p < 0.05) (Fig. 1B).

**Figure 2:**
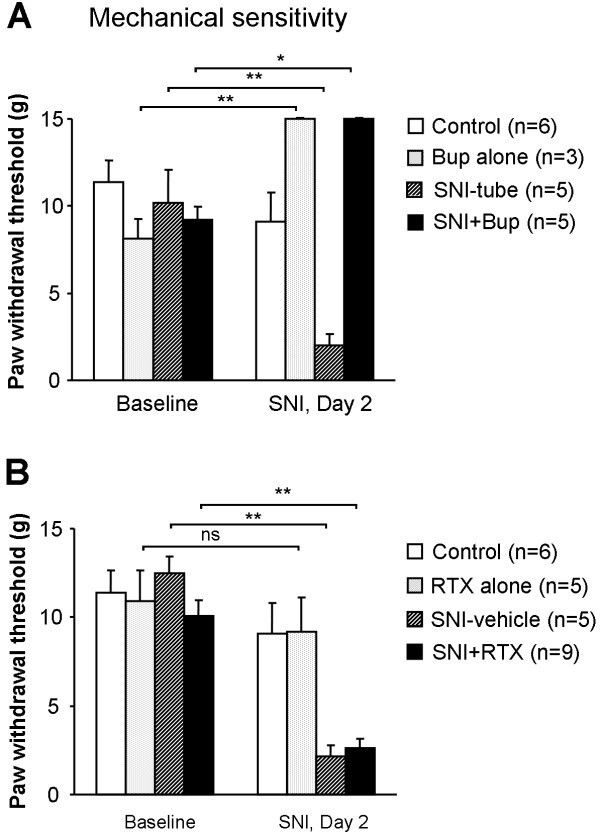
**Effects of nerve block with bupivacaine microspheres (Bup, A) and resiniferatoxin (RTX, B) on mechanical sensitivity before and two days after spared nerve injury (SNI)**. SNI-induced mechanical allodynia, i.e. decrease in paw withdrawal threshold, is not prevented by RTX treatment. Baseline pain sensitivity was tested before drug treatment and nerve injury *p < 0.05, **p < 0.01.

Two days after SNI, animals in the SNI-tube and SNI-vehicle groups also developed mechanical allodynia with a reduction of paw withdrawal thresholds from 10.2 and 12.5 g to 1.98 and 0.70 g, respectively (p < 0.01). In the bupivacaine (Bup)-treated groups (SNI+Bup) and Bup alone), the maximal value (15 g) was reached, indicating a complete blockade (Fig. 2A). In sharp contrast, RTX did not change the withdrawal threshold in animals without SNI (10.9 g vs 9.1 g, p > 0.05). Neither did RTX affect the development of mechanical allodynia in the SNI animals (Fig. 2B).

### SNI induces proliferation of microglia in the spinal cord

**Figure 3:**
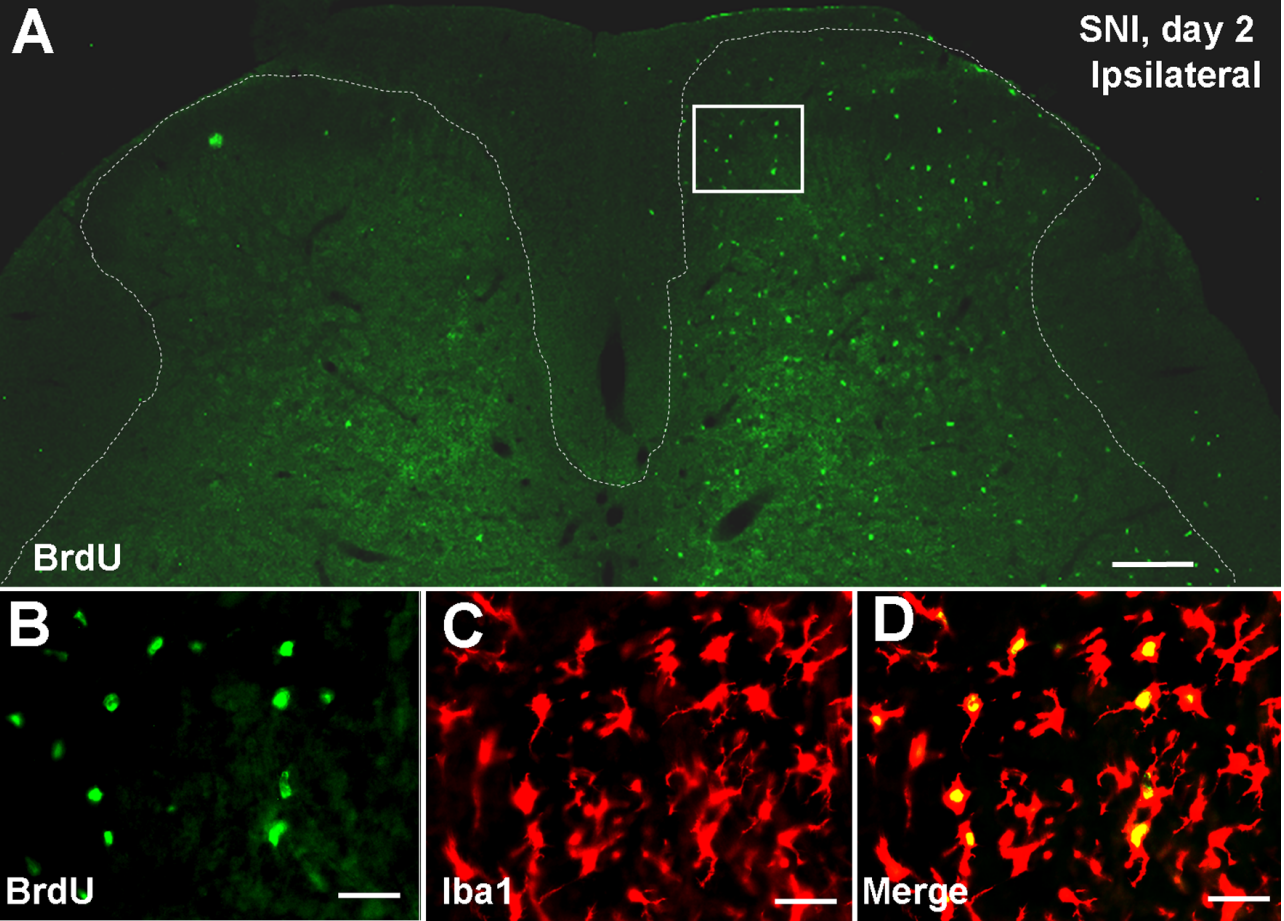
**SNI induces microglial proliferation in the spinal cord**. **(A) **Bromodeoxyuridine (BrdU) staining in the dorsal horn of the spinal cord two days after SNI. There is a dramatic increase in number of BrdU-positive profiles in the dorsal horn ipsilateral to nerve injury. Scale bar, 200 μm. **(B-D) **Enlargement of the ipsilateral medial dorsal horn (square indicated in A) showing co-localization of BrdU with the microglial marker Iba1. Scale bars, 50 μm.

**Figure 4:**
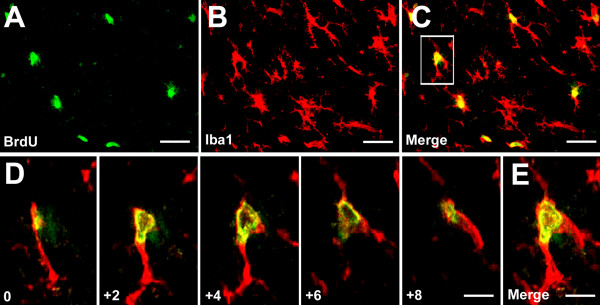
**Confocal microscopy images show BrdU expression in spinal microglia two days after SNI**. **(A-C) **Colocalization of BrdU and Iba1 in the medial superficial spinal cord. Scale bars, 50 μm. **(D) **Stack of confocal images (2 μm apart) from a double-labeled cell enlarged from a square in C. **(E) **Merge of all images in D. Scale bars, 20 μm.

To examine SNI-induced cell proliferation, we carried out bromodeoxyuridine (BrdU) immunostaining in the spinal cord. Two days after SNI, we observed a dramatic increase in the number of BrdU-positive cells in the dorsal horn of the spinal cord on the ipsilateral side of the nerve injury (53.8 ± 4.3), compared to that of the contralateral side (1.4 ± 0.3, p < 0.001) (Fig. 3A). Remarkably, almost all BrdU positive cells were colocalized with Iba1, a microglial marker (Fig. 3B-D). High-resolution images from confocal microscopy further confirmed that BrdU was only expressed in microglia (Fig. 4).

### Bupivacaine but not RTX inhibits microglial proliferation after SNI

**Figure 5:**
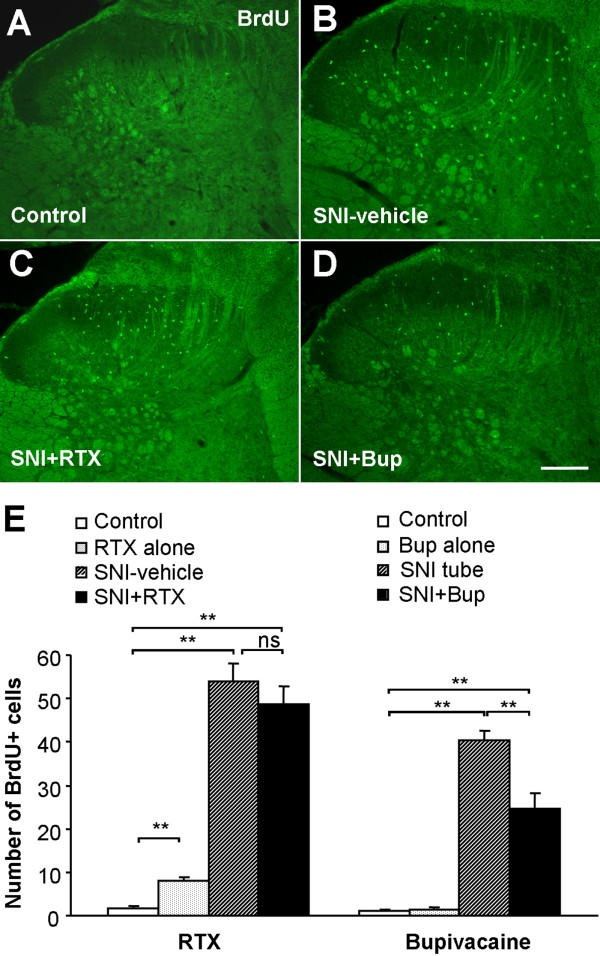
**Bupivacaine but not resiniferatoxin reduces cell proliferation in the spinal cord dorsal horn after SNI**. **(A-D) **BrdU immunostaining in the dorsal horn of control rats (A) and SNI rats receiving vehicle (B), RTX (C), and bupivacaine (Bup, D). **(E) **Number of BrdU-positive cell profiles in the dorsal horn: *Left*, effects of RTX on SNI-induced cell proliferation. *Right*: effects of bupivacaine on SNI-induced cell proliferation *p < 0.05, **p < 0.01, ns = no significance. Scale bar 200 μm. n = 3-5.

In the control group without SNI, there were only very few BrdU-positive cells in the dorsal horn (1.1 ± 0.1 for the tube control, and 2.4 ± 0.6 for the vehicle control, Fig. 5). Two days after SNI, there were 40.4 ± 2.1 and 53.8 ± 4.3 BrdU-positive cells in the vehicle and tube control group, respectively. RTX had no effect on SNI-induced microglial proliferation. The numbers of BrdU-positive cells in the SNI+RTX group (48.6 ± 4.3) and in the SNI-vehicle group (53.8 ± 4.3) were comparable (p = 0.51). In contrast, bupivacaine significantly inhibited microglial proliferation: the number of BrdU-positive cells in the SNI+Bup group and SNI-tube group was 24.5 ± 3.7 and 40.4 ± 2.1, respectively (n = 3, p < 0.05) (Fig. 5). RTX alone induced a slight but significant increase, from 2.4 ± 0.6 to 8.0 ± 0.8, in the number of BrdU-positive cells (p < 0.01). This increase may result from a mild nerve injury caused by RTX application.

### Bupivacaine but not RTX reduces p38 activation in the spinal cord after SNI

**Figure 6:**
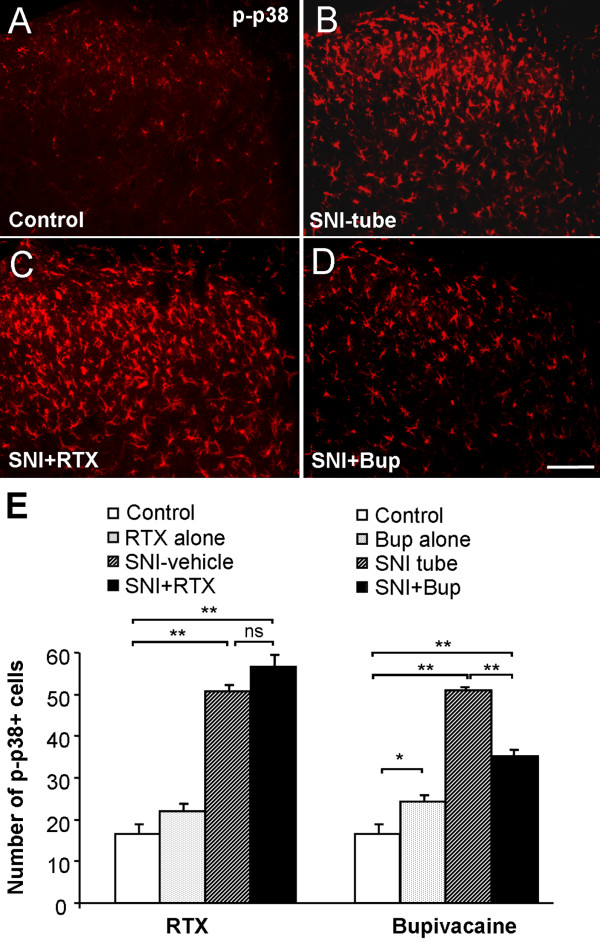
**Bupivacaine but not resiniferatoxin reduces the phosphorylation of p38 MAPK in the spinal cord dorsal horn after SNI**. **(A-D) **p-p38 immunostaining in the dorsal horn of control rats (A) and SNI rats receiving vehicle (B), RTX (C), and bupivacaine (Bup, D). **(E) **Number of p-p38-positive cell profiles in the dorsal horn: *Left*, effects of RTX on SNI-induced cell proliferation. *Right*: effects of bupivacaine on SNI-induced cell proliferation *p < 0.05, **p < 0.01, ns = no significance. Scale bar 100 μm. n = 3-5.

To investigate the SNI-induced activation of p38 MAPK in the spinal cord, we performed immunofluorescence using a phosphorylated-p38 (p-p38) antibody. Two days after SNI, a marked p38 activation was found in the dorsal horn of the spinal cord, and the number of p-p38-positive cells was 50.8 ± 1.5 in the SNI-vehicle group (vs 20.3 ± 2.2 in the control group, p < 0.0001) and 50.9 ± 0.7 in the SNI-tube group (vs 12.9 ± 2.3 in the control group, p < 0.0001) (Fig. 6). RTX had no effect on SNI-induced p-p38 increase: the numbers of p-p38-positive cells in the SNI-RTX group (56.6 ± 3.0) and in the SNI-vehicle group (50.8 ± 1.5) were comparable (p = 0.25). However, bupivacaine treatment markedly inhibited p38 activation, and the number of p-p38-positive cells decreased from 50.9 ± 0.7 in the SNI-tube group to 35 ± 1.7 in the SNI+Bup group (n = 3, p < 0.01). The Bup alone group also showed a mild but significant increase in p38 activation compared to the control group (p < 0.05).

## Discussion

We have made several interesting findings in the present study. First, SNI induced a marked proliferation of spinal cord microglia in the induction phase of neuropathic pain (day 2). Second, RTX specifically blocked the function of nociceptive fibers without affecting propriocetive and motor function. Third, SNI-induced microglial proliferation and p38 activation in the spinal cord was reduced by a complete sciatic nerve blockade with bupivacaine microspheres but not by a selective blockade of nociceptive fibers with RTX. Finally, RTX blocked heat hyperalgesia but failed to reduce mechanical allodynia after SNI.

### Microglial proliferation and activation in neuropathic pain

We found a substantial increase in the number of BrdU-positive cells in the ipsilateral dorsal horn 2 days after SNI. SNI-induced proliferation was restricted to Iba1-positive microglial cells. Our data are in agreement with other studies showing microglial proliferation after sciatic nerve transection [39] and sciatic nerve constriction [21] in rats and partial sciatic nerve ligation in mice [40]. Proliferation of spinal astrocytes has been shown after spinal nerve ligation in mice, using different protocol of bromodeoxyuridine injection [41]. Microglial activation can be described in different ways (morphologic, specific markers or proliferation) [17]. Unfortunately, many studies defined microglial activation only based on altered expression of Iba1 or OX-42 (CD11b), which lead to controversies when trying to link microglial activation and pain states, because the direct role of Iba1 and CD11b in neuropathic pain genesis is unclear. Indeed, several studies show that CD11b expression is not associated with pain states [42-45]. Thus, it is important to use additional markers such as ATP receptor P2X4 or active p38 MAPK that are functionally linked to neuropathic pain development [23,44]. We chose p-p38 as a marker of microglial activation 2 days after SNI, since at this time point p-p38 is specifically expressed in microglia [25]. To date there is no direct evidence that proliferation itself is necessary for the development of neuropathic pain, but it is reasonable to assume that an increase in the number of microglial cells will be associated with an increase in the production of pronociceptive mediators in microglia.

### Nociceptive-specific nerve conduction block

We have shown that a complete long-term nerve block using bupivacaine microspheres does not prevent neuropathic pain development in the SNI model after initial blockade wears off [32]. However during the period of the transmission block, the apoptosis of dorsal horn neurons [46] and the activation of p38 in microglia [25] are reduced. It appears that bupivacaine microspheres only delayed the development of pathophysiological events leading to neuropathic pain. In contrast, Xie et al. [6] have shown that neuropathic pain in the same model can be prevented by application of crystallized bupivicaine to the injury site (200 mg of a depot form bupivacaine). This discrepancy may result from different duration of nerve block. However, a complete nerve blockade for a very long period is not practical for patients because of impaired motor function.

TRPV1 is selectively expressed in C- and Aδ-nociceptive fibers [27,47,48]. Clinical studies using TRPV1 antagonists suffer from significant side effects such as hyperthermia [49]. Development of TRPV1 agonist (e.g., capsaicin or resiniferatoxin)-based pain therapy appears to be an attractive alternative [50]. TRPV1 agonists induce desensitization and inactivation of nociceptive neurons, impeding these neurons to respond to a subsequent stimulus [28]. Recent studies have shown that activation of TRPV1 with capsaicin or lidocaine can selectivity deliver a quaternary local anesthetic QX-314 to target only nociceptive fibers for a sustained blockade [26,30]. However the duration of this specific blockade (<12 h) may not be long enough for the purpose of the present study. RTX is an ultra potent agonist of TRPV1. Compared to capsaicin, RTX produces a shorter period of intense excitation but a longer period of desensitization [29]. After application of 0.01% RTX to the sciatic nerve, we found no impairment of motor function but a blockade of heat responses in naïve and SNI animals. However, SNI-induced tactile allodynia was not affected by RTX, in agreement with a previous study showing that stimulus-evoked tactile allodynia after nerve injury requires large Aβ-fibers [51].

### Primary afferent input, microglial activation, and neuropathic pain

Nerve injury-induced spontaneous activity is critical for the genesis of neuropathic pain. This spontaneous activity is also important for the early phase of p38 activation in spinal microglia [25] and OX-42 expression [52] in the spinal cord. But the relative contribution of C-fibers vs A-fibers to microglial activation remains elusive. A recent study shows that electrical stimulation at C-fiber intensity (10 mA, 5 min, 10 Hz) is sufficient to induce spinal Iba1 expression and mechanical allodynia without producing nerve injury. However, microglial activation by electrical stimulation, in the absence of nerve injury, is very mild. Indeed specific activation of C-fibers with capsaicin fails to activate spinal microglia [31]. Given the fact that electrical stimulation at C-fiber intensity will automatically activate A-fibers, the contribution of A-fibers to microglial activation cannot be excluded, although activation of A-fibers per se may not be sufficient to activate microglia.

In the spinal nerve ligation model, early ectopic activity in the first days is seen in myelinated fibers of the injured spinal nerve. This activity is also found in C-fibers of the neighbouring intact spinal nerve in a later stage (several weeks) [7,8,53,54]. However, Wu et al. reported C-fiber activity in neighbouring intact fibers in the first day [5]. After complete transection of the sciatic nerve, C-fibers show spontaneous activity one month after injury [55], whereas A-fibers exhibit this activity in the first several days [10]. Following partial sciatic transection, ectopic bursting discharges from Aδ- and C-fibers were recorded on post-injury day14-18 [56]. In the CCI model spontaneous activity is recorded primarily in myelinated fibers [57]. Although there is a burst of discharge in C fibers within minutes after injury, this burst fades after 15 minutes [12]. Therefore, spontaneous activity in early period after nerve injury is mainly fired in myelinated fibers, despite its presence in C-fibers. Consistently, we found that C-fibers had no role in early phase activation of microglia and development of mechanical allodynia in the SNI model.

Our data show that RTX fails to inhibit SNI-induced microglial proliferation and p38 activation in the spinal cord, suggesting that a selective nociceptive block (primarily targeting C-fibers) is not sufficient to prevent microglial activation. As the bupivacaine block (targeting all fibers) significantly reduced microglial activation, we conclude that activity of thicker myelinated A-fibers is required for microglial activation. However, since we are not able to specifically block the activity of large A-fibers, we cannot exclude a role of C-fibers in microglial activation. There are other possibilities that we should consider. First, TRPV1 is not expressed in all nociceptive fibers and therefore some nociceptive input could still reach to the spinal cord. Second, the C-fiber conduction block induced by RTX may not be complete. Third, the initial excitation triggered by RTX or mild nerve injury induced by RTX could induce microglial activation.

Our data also suggest that p38 activation in spinal microglia is associated with the development of mechanical allodynia but not heat hyperalgesia, because RTX blocks heat hyperalgesia but has no effect on microglial p38 activation and mechanical allodynia after SNI. Consistently, intrathecal injection of p38 inhibitors can effectively reduce mechanical allodynia after nerve injury [23-25,58] and paw incision [59]. In parallel, robust mechanical allodynia is also induced after spinal injection of ATP-activated microglia [44] or following activation of microglia by electrical stimulation [31]. In contrast, p38 activation in primary sensory neurons in the dorsal root ganglion is associated with the development of heat hyperalgesia by regulating the expression of TRPV1 [27,60].

Although bupivacaine blockade significantly reduced the microglial activation, it did not abolish the activation. The remaining microglial activation could be triggered by ectopic impulses arising proximally to the block, such as DRG and dorsal root. There is also activity-independent activation of microglia that is caused by nerve injury-induced disruption of retrograde axonal transport [61-64]. Glial cells may also activate each other by releasing pro-inflammatory cytokines and chemokines.

## Conclusion

We have demonstrated that microglial activation in the spinal cord after nerve injury is associated with the development of tactile allodynia, a cardinal feature of chronic pain. We have also revealed that a specific nociceptive blockade by RTX is insufficient to inhibit microglial activation and mechanical allodynia after SNI. Because bupivacaine can inhibit SNI-induced microglial activation, we conclude that spinal microglial activation after peripheral nerve injury requires peripheral spontaneous activity from large myelinated (Aβ/Aδ) afferent fibers, although we should not exclude the role of small unmyelinated (C) fibers. It is becoming obvious that major surgeries such as thoracotomy cause high incidence of chronic pain (neuropathic pain) due to possible nerve damage [1]. Given the importance of microglial activation in the development of neuropathic pain, the failure of nociceptive blockade to prevent microglial activation would indicate that this treatment will also fail to prevent the development of neuropathic pain in patients with major surgeries, although nociceptive blockade is effective to block acute pain. Indeed, blocking peripheral activity after surgery for acute pain relief and preventing the occurrence of chronic postoperative pain has shown variable results [65,66]. Additional treatment such as p38 MAP kinase inhibitor is needed to abolish or reduce microglial activation for the prevention of neuropathic pain development.

## Competing interests

The authors declare that they have no competing interests.

## Authors' contributions

MRS participated in experimental design, conducted nerve block, behavioral studies, histochemical studies and data analysis, and prepared the first draft of the manuscript. TB prepared confocal images. YJG participated in data analysis. ID participated in experimental design and made critical comments on the manuscript. RRJ designed the experiments, coordinated the project, and prepared the final version of the manuscript. All the authors read and approved the final manuscript.

## References

[B1] Kehlet H, Jensen TS, Woolf CJ (2006). Persistent postsurgical pain: risk factors and prevention. Lancet.

[B2] Kelly DJ, Ahmad M, Brull SJ (2001). Preemptive analgesia I: physiological pathways and pharmacological modalities. Can J Anaesth.

[B3] Devor M, Seltzer Z, Wall PD, Melzack R (1999). Pathophysiology of damaged nerves in relation to chronic pain. Textbook of Pain.

[B4] Ma C, Shu Y, Zheng Z, Chen Y, Yao H, Greenquist KW, White FA, LaMotte RH (2003). Similar electrophysiological changes in axotomized and neighboring intact dorsal root ganglion neurons. J Neurophysiol.

[B5] Wu G, Ringkamp M, Hartke TV, Murinson BB, Campbell JN, Griffin JW, Meyer RA (2001). Early onset of spontaneous activity in uninjured C-fiber nociceptors after injury to neighboring nerve fibers. J Neurosci.

[B6] Xie W, Strong JA, Meij JT, Zhang JM, Yu L (2005). Neuropathic pain: early spontaneous afferent activity is the trigger. Pain.

[B7] Han HC, Lee DH, Chung JM (2000). Characteristics of ectopic discharges in a rat neuropathic pain model. Pain.

[B8] Liu X, Eschenfelder S, Blenk KH, Janig W, Habler H (2000). Spontaneous activity of axotomized afferent neurons after L5 spinal nerve injury in rats. Pain.

[B9] Michaelis M, Liu X, Janig W (2000). Axotomized and intact muscle afferents but no skin afferents develop ongoing discharges of dorsal root ganglion origin after peripheral nerve lesion. J Neurosci.

[B10] Seburn KL, Catlin PA, Dixon JF, Lee MH, Matteson MS, Cope TC (1999). Decline in spontaneous activity of group Aalphabeta sensory afferents after sciatic nerve axotomy in rat. Neurosci Lett.

[B11] Tal M, Eliav E (1996). Abnormal discharge originates at the site of nerve injury in experimental constriction neuropathy (CCI) in the rat. Pain.

[B12] Blenk KH, Janig W, Michaelis M, Vogel C (1996). Prolonged injury discharge in unmyelinated nerve fibres following transection of the sural nerve in rats. Neurosci Lett.

[B13] Zimmermann M (2001). Pathobiology of neuropathic pain. Eur J Pharmacol.

[B14] Michaelis M, Vogel C, Blenk KH, Janig W (1997). Algesics excite axotomised afferent nerve fibres within the first hours following nerve transection in rats. Pain.

[B15] Sun Q, Tu H, Xing GG, Han JS, Wan Y (2005). Ectopic discharges from injured nerve fibers are highly correlated with tactile allodynia only in early, but not late, stage in rats with spinal nerve ligation. Exp Neurol.

[B16] Ji RR, Suter MR (2007). p38 MAPK, microglial signaling, and neuropathic pain. Mol Pain.

[B17] Suter MR, Wen YR, Decosterd I, Ji RR (2007). Do glial cells control pain?. Neuron Glia Biol.

[B18] Tsuda M, Inoue K, Salter MW (2005). Neuropathic pain and spinal microglia: a big problem from molecules in "small" glia. Trends Neurosci.

[B19] Hanisch UK, Kettenmann H (2007). Microglia: active sensor and versatile effector cells in the normal and pathologic brain. Nat Neurosci.

[B20] Horner PJ, Power AE, Kempermann G, Kuhn HG, Palmer TD, Winkler J, Thal LJ, Gage FH (2000). Proliferation and differentiation of progenitor cells throughout the intact adult rat spinal cord. J Neurosci.

[B21] Echeverry S, Shi XQ, Zhang J (2008). Characterization of cell proliferation in rat spinal cord following peripheral nerve injury and the relationship with neuropathic pain. Pain.

[B22] Graeber MB, Tetzlaff W, Streit WJ, Kreutzberg GW (1988). Microglial cells but not astrocytes undergo mitosis following rat facial nerve axotomy. Neurosci Lett.

[B23] Jin SX, Zhuang ZY, Woolf CJ, Ji RR (2003). p38 mitogen-activated protein kinase is activated after a spinal nerve ligation in spinal cord microglia and dorsal root ganglion neurons and contributes to the generation of neuropathic pain. J Neurosci.

[B24] Tsuda M, Mizokoshi A, Shigemoto-Mogami Y, Koizumi S, Inoue K (2004). Activation of p38 mitogen-activated protein kinase in spinal hyperactive microglia contributes to pain hypersensitivity following peripheral nerve injury. Glia.

[B25] Wen YR, Suter MR, Kawasaki Y, Huang J, Pertin M, Kohno T, Berde CB, Decosterd I, Ji RR (2007). Nerve Conduction Blockade in the Sciatic Nerve Prevents but Does Not Reverse the Activation of p38 Mitogen-activated Protein Kinase in Spinal Microglia in the Rat Spared Nerve Injury Model. Anesthesiology.

[B26] Binshtok AM, Bean BP, Woolf CJ (2007). Inhibition of nociceptors by TRPV1-mediated entry of impermeant sodium channel blockers. Nature.

[B27] Ji RR, Samad TA, Jin SX, Schmoll R, Woolf CJ (2002). p38 MAPK activation by NGF in primary sensory neurons after inflammation increases TRPV1 levels and maintains heat hyperalgesia. Neuron.

[B28] Szallasi A, Blumberg PM (1999). Vanilloid (Capsaicin) receptors and mechanisms. Pharmacol Rev.

[B29] Kissin I (2008). Vanilloid-induced conduction analgesia: selective, dose-dependent, long-lasting, with a low level of potential neurotoxicity. Anesth Analg.

[B30] Suzuki S, Gerner P, Colvin AC, Binshtok AM (2009). C-fiber-Selective Peripheral Nerve Blockade. The Open Pain Journal.

[B31] Hathway GJ, Vega-Avelaira D, Moss A, Ingram R, Fitzgerald M (2009). Brief, low frequency stimulation of rat peripheral C-fibres evokes prolonged microglial-induced central sensitization in adults but not in neonates. Pain.

[B32] Suter MR, Papaloizos M, Berde CB, Woolf CJ, Gilliard N, Spahn DR, Decosterd I (2003). Development of neuropathic pain in the rat spared nerve injury model is not prevented by a peripheral nerve block. Anesthesiology.

[B33] Kissin I, Davison N, Bradley EL (2005). Perineural resiniferatoxin prevents hyperalgesia in a rat model of postoperative pain. Anesth Analg.

[B34] Decosterd I, Woolf CJ (2000). Spared nerve injury: an animal model of persistent peripheral neuropathic pain. Pain.

[B35] Hu D, Hu R, Berde CB (1997). Neurologic evaluation of infant and adult rats before and after sciatic nerve blockade. Anesthesiology.

[B36] Thalhammer JG, Vladimirova M, Bershadsky B, Strichartz GR (1995). Neurologic evaluation of the rat during sciatic nerve block with lidocaine. Anesthesiology.

[B37] Chaplan SR, Bach FW, Pogrel JW, Chung JM, Yaksh TL (1994). Quantitative assessment of tactile allodynia in the rat paw. J Neurosci Methods.

[B38] Hargreaves K, Dubner R, Brown F, Flores C, Joris J (1988). A new and sensitive method for measuring thermal nociception in cutaneous hyperalgesia. Pain.

[B39] Liu L, Rudin M, Kozlova EN (2000). Glial cell proliferation in the spinal cord after dorsal rhizotomy or sciatic nerve transection in the adult rat. Exp Brain Res.

[B40] Narita M, Yoshida T, Nakajima M, Narita M, Miyatake M, Takagi T, Yajima Y, Suzuki T (2006). Direct evidence for spinal cord microglia in the development of a neuropathic pain-like state in mice. J Neurochem.

[B41] Xu M, Bruchas MR, Ippolito DL, Gendron L, Chavkin C (2007). Sciatic nerve ligation-induced proliferation of spinal cord astrocytes is mediated by kappa opioid activation of p38 mitogen-activated protein kinase. J Neurosci.

[B42] Colburn RW, DeLeo JA, Rickman AJ, Yeager MP, Kwon P, Hickey WF (1997). Dissociation of microglial activation and neuropathic pain behaviors following peripheral nerve injury in the rat. J Neuroimmunol.

[B43] Obata H, Eisenach JC, Hussain H, Bynum T, Vincler M (2006). Spinal glial activation contributes to postoperative mechanical hypersensitivity in the rat. J Pain.

[B44] Tsuda M, Shigemoto-Mogami Y, Koizumi S, Mizokoshi A, Kohsaka S, Salter MW, Inoue K (2003). P2X4 receptors induced in spinal microglia gate tactile allodynia after nerve injury. Nature.

[B45] Winkelstein BA, DeLeo JA (2002). Nerve root injury severity differentially modulates spinal glial activation in a rat lumbar radiculopathy model: considerations for persistent pain. Brain Res.

[B46] Scholz J, Broom DC, Youn DH, Mills CD, Kohno T, Suter MR, Moore KA, Decosterd I, Coggeshall RE, Woolf CJ (2005). Blocking caspase activity prevents transsynaptic neuronal apoptosis and the loss of inhibition in lamina II of the dorsal horn after peripheral nerve injury. J Neurosci.

[B47] Caterina MJ, Schumacher MA, Tominaga M, Rosen TA, Levine JD, Julius D (1997). The capsaicin receptor: a heat-activated ion channel in the pain pathway. Nature.

[B48] Ma QP (2001). Vanilloid receptor homologue, VRL1, is expressed by both A- and C-fiber sensory neurons. Neuroreport.

[B49] Gavva NR, Treanor JJ, Garami A, Fang L, Surapaneni S, Akrami A, Alvarez F, Bak A, Darling M, Gore A, Jang GR, Kesslak JP, Ni L, Norman MH (2008). Pharmacological blockade of the vanilloid receptor TRPV1 elicits marked hyperthermia in humans. Pain.

[B50] Knotkova H, Pappagallo M, Szallasi A (2008). Capsaicin (TRPV1 Agonist) therapy for pain relief: farewell or revival?. Clin J Pain.

[B51] Ossipov MH, Bian D, Malan TP, Lai J, Porreca F (1999). Lack of involvement of capsaicin-sensitive primary afferents in nerve-ligation injury induced tactile allodynia in rats. Pain.

[B52] Xie W, Strong JA, Zhang JM (2009). Early Blockade of Injured Primary Sensory Afferents Reduces Glial Cell Activation in Two Rat Neuropathic Pain Models. Neuroscience.

[B53] Ali Z, Ringkamp M, Hartke TV, Chien HF, Flavahan NA, Campbell JN, Meyer RA (1999). Uninjured C-fiber nociceptors develop spontaneous activity and alpha-adrenergic sensitivity following L6 spinal nerve ligation in monkey. J Neurophysiol.

[B54] Djouhri L, Koutsikou S, Fang X, McMullan S, Lawson SN (2006). Spontaneous pain, both neuropathic and inflammatory, is related to frequency of spontaneous firing in intact C-fiber nociceptors. J Neurosci.

[B55] Govrin-Lippmann R, Devor M (1978). Ongoing activity in severed nerves: source and variation with time. Brain Res.

[B56] Pan HL, Eisenach JC, Chen SR (1999). Gabapentin suppresses ectopic nerve discharges and reverses allodynia in neuropathic rats. J Pharmacol Exp Ther.

[B57] Kajander KC, Bennett GJ (1992). Onset of a painful peripheral neuropathy in rat: a partial and differential deafferentation and spontaneous discharge in A beta and A delta primary afferent neurons. J Neurophysiol.

[B58] Svensson CI, Fitzsimmons B, Azizi S, Powell HC, Hua XY, Yaksh TL (2005). Spinal p38beta isoform mediates tissue injury-induced hyperalgesia and spinal sensitization. J Neurochem.

[B59] Wen YR, Suter MR, Ji RR, Yeh GC, Wu YS, Wang KC, Kohno T, Sun WZ, Wang CC (2009). Activation of p38 Mitogen-activated Protein Kinase in Spinal Microglia Contributes to Incision-induced Mechanical Allodynia. Anesthesiology.

[B60] Obata K, Yamanaka H, Kobayashi K, Dai Y, Mizushima T, Katsura H, Fukuoka T, Tokunaga A, Noguchi K (2004). Role of mitogen-activated protein kinase activation in injured and intact primary afferent neurons for mechanical and heat hypersensitivity after spinal nerve ligation. J Neurosci.

[B61] Abe N, Cavalli V (2008). Nerve injury signaling. Curr Opin Neurobiol.

[B62] Perlson E, Hanz S, Ben-Yaakov K, Segal-Ruder Y, Seger R, Fainzilber M (2005). Vimentin-dependent spatial translocation of an activated MAP kinase in injured nerve. Neuron.

[B63] Cavalli V, Kujala P, Klumperman J, Goldstein LS (2005). Sunday Driver links axonal transport to damage signaling. J Cell Biol.

[B64] O'Brien JJ, Nathanson NM (2007). Retrograde activation of STAT3 by leukemia inhibitory factor in sympathetic neurons. J Neurochem.

[B65] Senturk M, Ozcan PE, Talu GK, Kiyan E, Camci E, Ozyalcin S, Dilege S, Pembeci K (2002). The effects of three different analgesia techniques on long-term postthoracotomy pain. Anesth Analg.

[B66] Nikolajsen L, Ilkjaer S, Christensen JH, Kroner K, Jensen TS (1997). Randomised trial of epidural bupivacaine and morphine in prevention of stump and phantom pain in lower-limb amputation. Lancet.

